# Nucleated red blood cells as early indicators of sepsis in severe burns

**DOI:** 10.1093/burnst/tkaf075

**Published:** 2025-11-24

**Authors:** Feras Almujalli, Ali Asiri, Jon Hazeldine, Jon Bishop, Naiem S Moiemen, Paul Harrison

**Affiliations:** Department of Inflammation and Ageing, School of Infection, Inflammation and Immunology, College of Medicine and Health, University of Birmingham, Birmingham, B15 2TT, UK; Department of Inflammation and Ageing, School of Infection, Inflammation and Immunology, College of Medicine and Health, University of Birmingham, Birmingham, B15 2TT, UK; Department of Inflammation and Ageing, School of Infection, Inflammation and Immunology, College of Medicine and Health, University of Birmingham, Birmingham, B15 2TT, UK; Department of Inflammation and Ageing, School of Infection, Inflammation and Immunology, College of Medicine and Health, University of Birmingham, Birmingham, B15 2TT, UK; Department of Inflammation and Ageing, School of Infection, Inflammation and Immunology, College of Medicine and Health, University of Birmingham, Birmingham, B15 2TT, UK; Department of Inflammation and Ageing, School of Infection, Inflammation and Immunology, College of Medicine and Health, University of Birmingham, Birmingham, B15 2TT, UK

**To the Editor**,

Sepsis is a leading cause of morbidity and mortality in patients with severe burns, yet its early recognition remains a major clinical challenge. Conventional haematological indices provide limited prognostic information, highlighting the need for simple and objective biomarkers that can support risk stratification. Nucleated red blood cells (NRBCs), absent in the circulation of healthy adults, appear during severe hypoxia, systemic inflammation, and bone marrow stress [1]. Although NRBCs have been associated with poor outcomes in critical illness, their predictive role in burns and sepsis has not been clearly defined.

We conducted a prospective cohort study of 96 adults with ≥20% total body surface area (TBSA) burns admitted to the major burns unit at the Queen Elizabeth Hospital, Birmingham (median age 49 years, median %TBSA 32%, 41% with inhalation injury). Peripheral blood samples were collected on days 1–14, day 28, and at 3, 6, and 12 months post-injury. Clinical data, including sepsis status, were prospectively recorded. Sepsis was defined according to the 2007 American Burn Association (ABA) consensus criteria, which remain widely applied in burns research [2].

Blood samples were analysed using the Sysmex XN-1000 haematology analyser. The instrument underwent daily internal quality control with manufacturer reagents and was enrolled in the UK National External Quality Assessment Scheme (UKNEQAS), ensuring reproducibility. Standard red cell parameters [red blood cell (RBC) count, haemoglobin (HGB), haematocrit (HCT), mean corpuscular HGB concentration] were measured alongside fragmented red cells (FRCs) and NRBC counts.

**Figure 1:**
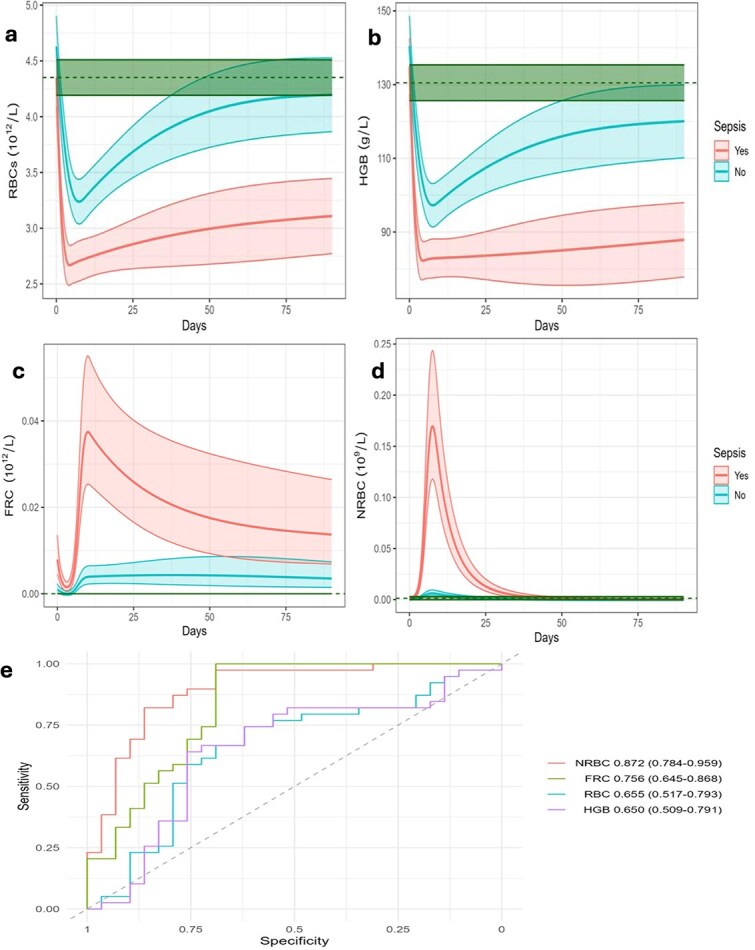
Time-course and predictive analysis of red cell indices and NRBCs in septic versus non-septic burn patients. (a) RBC counts, (b) HGB concentrations (HGB), (c) FRC, and (d) NRBCs, shown across all sampling time points. Data are presented as median ± interquartile range. Septic patients exhibited lower HGB, RBC, and FRC values compared with non-septic patients, while NRBCs emerged between days 4 and 14 and were significantly higher in septic patients. (e) Receiver operating characteristic (ROC) curves at day 7 comparing the discriminatory ability of NRBCs, FRCs, RBC counts, and HGB for sepsis. Figure labels show AUROC values with 95% confidence intervals. *NRBCs* nucleated red blood cells, *RBC* red blood cell, *HGB* haemoglobin, *FRCs* fragmented red cells, *ROC* receiver operating characteristic

On day 1, HGB, HCT, and RBC counts were elevated, reflecting early haemoconcentration caused by plasma loss and capillary leak before full fluid resuscitation. FRCs also peaked at this time, consistent with direct burn-related red cell damage. In the early post-injury phase (days 2–3), HGB, HCT, and RBC counts declined sharply due to haemodilution, haemolysis, and erythrocyte fragmentation in our cohort. Septic patients experienced greater and more prolonged reductions in HGB, HCT, and RBC count compared with non-septic patients, with delayed recovery beyond day 14 (Figure 1a,b). Similar trends have been reported previously [3, 4]. FRCs remained higher in septic patients compared with non-septic patients, with a second peak around day 7 (Figure 1c). NRBCs appeared later, between days 4 and 14, with a median peak at day 7, coinciding with the period of highest sepsis incidence. Septic patients showed consistently higher NRBC counts than non-septic patients during this window (Figure 1d). On day 7, NRBCs predicted sepsis with an Area Under the Receiver Operating Characteristic curve (AUROC) of 0.872 (0.784–0.959), outperforming HGB, HCT, and RBC counts (AUROCs < 0.75 across comparable time points) (Figure 1e). This analysis was adjusted for potential confounders, including age, burn depth, and inhalation injury.

The biological mechanism for NRBC release is well established. Following severe burns, cytokines such as IL-6 and TNF-α rise sharply, triggering erythropoietin production but also blunting the marrow’s response to it, which is a process known as anaemia of inflammation. The resulting ineffective erythropoiesis and marrow stress, combined with tissue hypoxia, cause premature erythroblast release into circulation, explaining the NRBC surge observed in septic patients. In our study, this translated into strong prognostic value. NRBC emergence preceded or coincided with sepsis onset, serving as an early warning signal. Unlike complex assays, NRBCs can be measured rapidly and at no additional cost during routine full blood counts on modern haematology analysers, making them a practical biomarker in burn care.

Strengths include the prospective design, relatively large cohort, and intensive longitudinal sampling during the highest-risk period for sepsis. Rigorous internal and external quality assurance strengthens measurement reliability. Limitations include the single-centre design and the use of the ABA 2007 definition for sepsis diagnosis. Whilst Sepsis-3 criteria emphasize organ dysfunction, the ABA definition remains widely used in burns because it accounts for burn-specific physiology [2]. Future research should assess NRBC performance against Sepsis-3 for broader applicability.

In practice, appearance or a rising trend of NRBCs between days 4–14 post-injury should prompt immediate sepsis screening, microbiological cultures, and closer clinical review. Integrating automated NRBC alerts into laboratory reports would support timely intervention within routine workflows. Routine monitoring of NRBCs could enhance post-burn surveillance. Their appearance should alert clinicians to increased sepsis risk, prompting earlier investigation, closer monitoring, and pre-emptive interventions. As a marker available from routine haematology testing, NRBCs provide a low-cost, accessible tool for improving sepsis recognition. This aligns with [1], who found NRBCs associated with sepsis and poorer outcomes in burn patients.

In this prospective cohort of 96 adults with severe burns, NRBCs emerged between days 4 and 14 and provided strong early discrimination of patients who developed sepsis. With an AUROC of 0.872 (0.784–0.959) on day 7, NRBCs outperformed conventional red cell indices. These findings support integrating NRBC measurement into routine clinical monitoring as a simple, effective approach to earlier sepsis recognition. Multicentre validation and assessment against Sepsis-3 criteria are warranted.
